# Evaluating a pedagogical approach to promoting academic integrity in higher education: An online induction program

**DOI:** 10.3389/fpsyg.2022.1009305

**Published:** 2022-10-05

**Authors:** Laura Sbaffi, Xin Zhao

**Affiliations:** Information School, The University of Sheffield, Sheffield, United Kingdom

**Keywords:** academic integrity, policy and practice, educational games, online module, online pedagogy

## Abstract

Academic integrity is at the heart of excellent education. However, resources explaining the concept tend to be definition-driven, while using complex language and sometimes even an austere tone designed to discourage students from breaches. This study aims to design and evaluate an online module at a UK University across 2 years, designed to improve students’ understanding of concepts of academic integrity and practice. The module includes a range of interactive resources (e.g., gamified quizzes and e-booklets) and was made available to a large cohort of postgraduate students (448). The study adopts a mixed-methods approach composed of three sequential phases involving first collecting students’ views on existing academic integrity resources (7 students participating in a focus group and 39 competing a questionnaire), then developing a range of new ones based on students’ feedback to form the content of the module, and finally gathering students’ evaluation on the newly created resources (sample size: 361 students). Results illustrate a clear improvement in relation to the accessibility, usefulness and understandability of new resources. Results also highlight a remarkable increase in student confidence levels regarding academic integrity. Students also considered the new module as more appealing and informative. This manuscript offers a good example of a pedagogical approach aimed at promoting academic integrity in an innovative and engaging fashion.

## Introduction

Academic integrity is at the heart of excellent teaching and learning and can be defined as a commitment and demonstration of honest and moral behaviour in an academic setting and it is applicable to both students and academic staff. The lack of such commitment can lead to academic dishonesty, which is “related to the deterioration of educational goals, specifically ideas that impact learners’ intellectual, civic, and psychosocial development” (Eshet and Margaliot, 2022, p. 2). Macfarlane et al. (2014) conducted a systematic literature review showing that research into academic integrity is often centred on unethical student behaviours, which may be either accidental or intentional (Walker and White, 2014). For example, Greenberger et al. (2016) argued that one of the most common breaches of academic integrity is plagiarism, caused by poor paraphrasing practices and incorrect referencing formats. At the other end of the spectrum is the deliberate attempts at cheating, which range from buying, selling, or trading essays, to arranging for someone else to take an exam (Bretag et al., 2019; Awdry et al., 2021). However, research suggests that the definition of academic integrity is not universally understood and is open to different interpretations; this may cause misunderstanding among staff and students (Waltzer and Dahl, 2022), and ultimately lead to unethical academic behaviours (Gullifer and Tyson, 2014).

Upholding academic integrity in higher education is vital for all stakeholders. Nevertheless, universities face numerous challenges related to breaches in student academic integrity, whether unintentional or deliberate (Mahmud and Ali, 2021). A key issue appears to be centred on aligning concepts of academic integrity, policies and processes, with teaching and learning practices. This study aims to design, implement and evaluate an online academic integrity module with resources tailored to students’ needs. This manuscript presents results of qualitative and quantitative evaluation data, aiming to inform current teaching practice related to academic integrity in higher education.

### The challenges of student academic integrity development in higher education

Existing literature highlights many barriers to the teaching of academic integrity within the higher education sector. One significant barrier appears to be the complex terminology and unclear processes associated with breaches in academic integrity (Wong et al., 2016; Ransome and Newton, 2018; Bretag et al., 2019). Consequently, students may be left vulnerable to penalties due to inadequate understanding of academic integrity (Palmer et al., 2019). Another barrier is often caused by competing views across cultures of what constitutes academic integrity and unethical academic conduct (Zhang et al., 2014; Chien, 2017; Palmer et al., 2017; Kam et al., 2018; Khanal and Gaulee, 2019; Błachnio et al., 2021), or even across academic institutions within a single national culture (Bretag et al., 2014; Walker and White, 2014). This may result in mixed messages to students who enter a new university with misaligned prior knowledge of these concepts in relation to institutional requirements (Bertram Gallant, 2017). Other barriers that have been frequently mentioned in the literature include language (e.g., unfamiliarity with academic writing style) (Newton, 2016) and cultural barriers (Bista, 2011), which are often experienced by students whose first language is not English (Fass-Holmes, 2017; Jian et al., 2019). For example, Mahmud et al. (2019) highlighted significant differences in student perceptions of academic integrity in relation to the UK and Eastern European countries, advocating that academic integrity policies should be considerate of national cultures. Clough et al. (2015) interviewed 30 Unfair Means officers, finding that unfair means, or breaches of academic integrity in essay-based assignments, are more common among non-native English speakers. Other research highlighted a danger in the over-simplistic view that international students cheat due to culturally diverse values (Bretag et al., 2019), potentially leading to staff being biased towards them (Zhao and Kung, 2021).

### Promoting academic integrity in higher education

Recent research developments heightened the requirement for a holistic approach in promoting academic integrity, including the establishment of clearly defined academic integrity principles and terminologies. Löfström et al. (2015) observed that there are mismatches in the perspectives of teachers and students regarding responsibility for upholding academic integrity standards in universities. Bealle (2017) argued that students, as those most affected by academic integrity policies, tend to become passive recipients rather than active upholders. In the same vein, Bretag et al. (2014) called for universities to move beyond mere information provision on academic integrity to engage students by integrating education and support into their academic curriculum.

Existing research recognises two dominant approaches to preventing student academic integrity breaches: punitive and educative. Richards et al. (2016, p. 243) suggested that a punitive approach aims to “deter students from committing breaches through the threat of penalties,” whereas an educative approach aims to “reduce the likelihood of students committing breaches by providing them with relevant skills and knowledge.” Conversely, Walker and White (2014) proposed two plagiarism prevention models, the “ethical” model, emphasising students’ active role in adhering to the academic integrity code of conduct, and the “pedagogical model,” focused on equipping students with appropriate academic skills. Although a punitive approach can communicate to students that plagiarism has serious consequences, research indicates that this approach alone is not sufficient to reduce cheating (Miller et al., 2011; Sun and Hu, 2020). Zhao and Kung (2021) highlighted the importance for universities to adopt an educative (pedagogical) approach to provide consistent and continuous teaching for students related to academic integrity before applying a more severe punitive or judicial approach as more serious cases, such as contract cheating, are found. Similarly, Palmer et al. (2019) warned that overly punitive measures may not be effective at reducing academic integrity breaches at universities.

Following a pedagogical approach, a number of researchers have highlighted the value of an early intervention strategy that is positive, proactive, engaging and continuous to help students’ academic integrity development when entering a new department (Belter and Du Pré, 2009; Bista, 2011; Bretag et al., 2014; Walker and White, 2014; Newton, 2016). For example, Bertram Gallant (2017, p. 89) suggested that universities should shift the focus from enforcing rules and policies to ensuring students learn about academic integrity, fostering “a learning-oriented environment, improving instruction, and enhancing institutional support for teaching and learning.” Pàmies et al. (2020) argued that universities need to take more responsibility for educating their students about plagiarism and explaining how to properly cite sources.

Research suggests that there has been improvement in university commitment to addressing academic integrity issues (Burbidge and Hamer, 2020); however, the implementation process within teaching remains unsatisfactory (Bretag et al., 2014; Gottardello and Karabag, 2020). Christie et al. (2013) reported a disparity between academic integrity promotion and actual teaching practice which assimilates academic integrity in the classroom. Particularly, postgraduate students are less informed of academic integrity policies (Fatemi and Saito, 2020), and often left underprepared for research-based academic assignments (Mahmud and Bretag, 2013). Therefore, it is of vital importance to help students develop a clear understanding of academic integrity (Tatum and Schwartz, 2017).

A number of educative strategies have proven effective. For example, researchers have demonstrated that educational initiatives, such as online modules that focus on academic integrity, can positively impact students’ attitudes, reducing potentially unethical behaviours (Belter and Du Pré, 2009; Ballard, 2013; Bealle, 2017; Palmer et al., 2019; Sefcik et al., 2019; Du, 2020). Stephens et al. (2021) argued, however, that online courses are only partially useful for students, but they can become much more effective within a comprehensive approach to promoting academic integrity. Boehm et al. (2009) suggested that providing clear definitions with specific examples of what constitutes unethical behaviours can effectively prevent academic integrity breaches. Bretag et al. (2014) argued that education resources on academic integrity should be engaging and creative, for example by using storytelling and narrations. Furthermore, they suggested that regular email reminders should be incorporated, providing ongoing support for students. Macfarlane et al. (2014) proposed that the provision of educative resources, and design of student-centred activities, should be informed by student feedback and tailored towards student needs. However, relatively less empirical studies directly addressed the topic of academic integrity through the design and evaluation of interventional strategies, such as academic integrity courses (Elander et al., 2010; Cronan et al., 2017; Stoesz and Yudintseva, 2018; Perkins et al., 2020; Sotiriadou et al., 2020).

### Research aim and objectives

This research aims to design and evaluate a newly created module enhancing students’ understanding of academic integrity concepts, policies and practices. The module is designed to include a suite of interactive activities and engaging materials which are based on student feedback and suggestions.

The objectives of this research are:

• To explore students’ needs for academic integrity related activities and resources.
• To design and implement an online module with interactive activities and learning resources to support students’ academic integrity development based on student feedback.
• To examine the effectiveness of the online module in enhancing students’ awareness and knowledge of academic integrity.

## Materials and methods

### Research design

**FIGURE 1:**
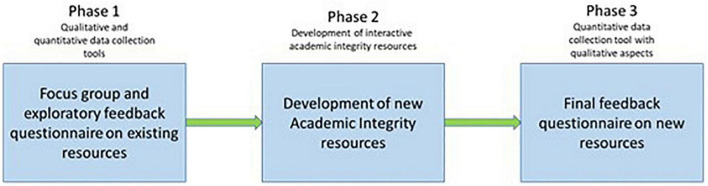
Phases of the study.

This study adopted a mixed-methods inductive approach and was conducted in a post-graduate school at a research-intensive university in the UK. Both quantitative and qualitative data were gathered across the course of a sequential study composed of three main phases (Figure 1).

In phase 1 (January-February 2020), a focus group interview and an online survey were conducted with postgraduate students within the Social Science faculty at a UK university to explore their views and understanding of academic integrity. In the focus group, in-depth discussions were carried out with a sample of seven students from different postgraduate programmes of study and genders. Students were asked questions around three main areas: their general understanding of the principles and concepts of academic integrity, the support received to date with respect to academic integrity, and their views on the existing academic integrity resources. The focus group interview questions had been previously piloted with two independent students to assess the clarity and relevance of the questions. Analysis of focus group discussions informed the design of an online questionnaire covering aspects related to student understanding of academic integrity, approaches to searching for academic integrity-related resources, their perception and needs in terms of content and style of the academic integrity resources available, and suggestions to make them more relevant and accessible. Furthermore, it listed eight aspects of existing academic integrity resources (reliable, comprehensive, easy to use, useful, credible, convenient and accessible, easy to understand and trustworthy) which students were asked to rate on a Likert scale from 1 (completely disagree) to 5 (completely agree). Cronbach’s Alpha (0.941) was used to measure the internal consistency of the eight aspects. The questionnaire was pre-tested for content validity by two expert colleagues in the field of Information Science. It was then piloted with three independent students for readability and coherence of questions and was distributed to postgraduate students within the Faculty of Social Science using the university volunteer student list.

**FIGURE 2:**
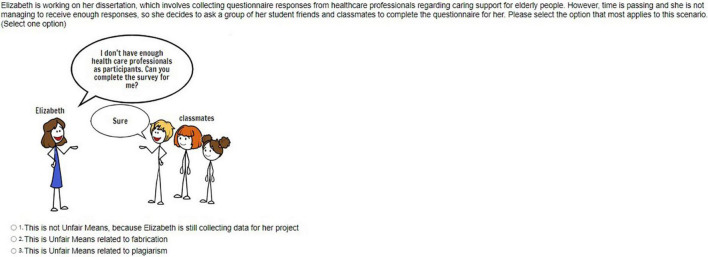
Pictured left is a representative example of a question included in the 20-question quiz; on the right is a screenshot of a page from the e-booklet.

In phase 2 (April-September 2020), the authors developed a range of resources based on student feedback from phase 1, aimed at enhancing accessibility, with more detailed content and support regarding academic integrity education within the department. These resources, collated under the term “Academic Integrity Activities,” were hosted in an online Blackboard module accessible to students. The package included an interactive video recording with embedded questions on academic integrity and other key information for students; a gamified academic integrity quiz with 20 scenario questions; a FAQ document related to common academic integrity-related queries; an electronic booklet (e-booklet) containing detailed examples of commonly occurring academic integrity problems and their solutions (see Figure 2 for screenshots examples of some of the resources). Students were asked to access and familiarise themselves with the resources and undertake the quiz as part of induction activities at the beginning of the academic year. Additionally, a series of four online, synchronous online sessions were offered to students, spread throughout the Autumn Semester; the sessions were student-led, covering topics suggested by students *via* online polls which informed the design and content of the next session.

In phase 3 (September-November 2020), a new online questionnaire was distributed to obtain students’ feedback regarding the newly created academic integrity resources. The questionnaire included, similarly to that of phase 1, the rating of eight key aspects of academic integrity and questions regarding the timeliness of support in the academic year, and in what ways the new resources could be further improved and promoted to students. Furthermore, respondents were asked about their confidence in relation to academic integrity-related concepts before and after use of the new resources. Phases 1 and 3 sought and received ethical approval from the University Research Ethics Committee.

### Participants

In phase 1, both focus group and online survey participants were postgraduate students from the Faculty of Social Science at a UK university. The reason for adopting this approach was twofold: first, to gain a deep understanding of the issues specifically faced by the students enrolled in the department under study (which is part of the Faculty of Social Science) and second, to acquire a wider appreciation of academic integrity perceptions at institutional level. The integration of the two perspectives would aid the formulation of truly comprehensive resources. Research was advertised *via* email; only volunteer students participated and no incentives were offered. The students participating in the focus group were four females and three males (mean age: 23 years), all holding international student status. The questionnaire from phase 1 was completed by 39 students. It was not possible to establish a response rate as the questionnaire was sent *via* the university volunteering mailing list. In this sample 2/3 of the students were female (66.7%) and almost half (46.2%), domestic students (Table 1) and had a mean age of 25 years. Participants in phase 3 were postgraduate students enrolled in varied programmes of study, but working within one department of the Faculty of Social Science; all accessed the induction module containing phase 2 induction activities. The students in these programmes learn in a hybrid environment offering a combination of online and face-to-face activities. Historically, however, academic integrity aspects are covered in an online format as they involve the whole student cohort and would be impractical to manage in other ways.

**TABLE 1:** Demographic characteristics of the students participating in the research.

	Phase 1 questionnaire (*N* = 39)	Phase 1 focus group (*N* = 8)	Phase 3 questionnaire (*N* = 130)
**Gender**			
Male	33.3% (13)	50.0% (4)	36.2% (47)
Female	66.7% (26)	50.0% (4)	60.8% (79)
Other/Prefer not to say	0.0% (0)	0.0% (0)	3.0% (4)
**Nationality**			
Domestic	46.2% (18)	0.0% (0)	25.4% (33)
European	10.2% (3)	25.0% (2)	4.6% (6)
Overseas	43.6% (17)	75.0% (6)	69.2% (90)
Prefer not to say	0.0% (0)	0.0% (0)	0.8% (1)
**Awareness of academic integrity concepts before joining the department**			
Yes	66.7% (26)	87.5% (7)	76.2% (99)
No	17.9% (7)	0.0% (0)	15.4% (20)
Not sure	15.4% (6)	12.5% (1)	8.4% (11)

The questionnaire was included as the last task for completion in the induction activities of the online module. The rationale behind the inclusion of only postgraduate students from an academic department was based on the fact all resources created in phase 2 were designed specifically for this cohort (e.g., examples and quizzes were designed with the specific academic writing requirements of the department). 130 students out of 448 returned the phase 3 feedback questionnaire (response rate 29%, mean age: 26 years). Similar to phase 1, the majority of respondents were female (60.8%). However, this time a higher proportion of overseas students (69.2%) completed the survey compared to phase 1.

### Research ethics

The project has received ethics approval (ID: 032172) from The University of Sheffield Ethics Committee. The approval letter has been uploaded as part of Supplementary material. An information sheet was provided to participants prior to data collection. Inform consent was collected prior to the survey and interviews. All data were anonymised by using a number system. Participants were reminded about the right to withdraw freely from the project.

### Data analysis

Descriptive statistics, independent sample *t*-tests and comparison of means, were used to analyse quantitative data derived from the two questionnaires using IBM SPSS 26. Qualitative data from the focus group, and open-ended questions from the two questionnaires, were manually transcribed and analysed using the six-step approach to thematic analysis established by Braun and Clarke (2006). Thematic analysis was adopted to support a deeper understanding of participants’ individual circumstances and experiences of academic integrity.

## Results

### Focus group

In phase 1, seven students participated in a focus group which highlighted three areas for improvements: academic integrity-related terminology, delivery formats, and tone of communication. Students found that the terminology around academic integrity tended to be complex and less engaging; they recommended that academic integrity information should include “real examples” with analysis, and “scenario questions” that are easy to follow and appear relatable:

“The definitions are ok, but I would have liked more examples, practical scenarios so that it’s easier to know what to avoid” (Focus group participant 1, China, Female)

They also recommended that the content of academic integrity should be delivered in a more engaging manner, suggesting interactive videos, humorous print/online brochures, and drop-in sessions for Q&A:

“Maybe print brochures and include them in the welcome pack, or draw some comics/make short videos (better if humorous)” (Focus group participant 5, China, Female)

Additionally, students found that the tone of academic integrity related communications tended to be “scary.” They expressed a preference towards a more neutral or supportive tone:

“I hope there could be a better way to approach this issue, such as the officer should hold a neutral ground and try to guide and explain to students of their mistakes and not try to make them feel ashamed of what happened” (Focus group participant 3, Singapore, Male)

### Pre and post-intervention quantitative data analysis

Two thirds of the students who completed the phase 1 questionnaire were aware of academic integrity concepts prior to their arrival at university. However, the remaining ones did not, or were unsure. In phase 3, 361 postgraduate students from one academic department completed academic integrity activities, representing a completion rate of 81%. Of those students, 130 completed the feedback questionnaire (Table 1). In terms of initial awareness of concepts of academic integrity, 76.2% reported a clear understanding prior to joining the department. Of those reporting no awareness of academic integrity, 74% were non-domestic students.

**FIGURE 3:**
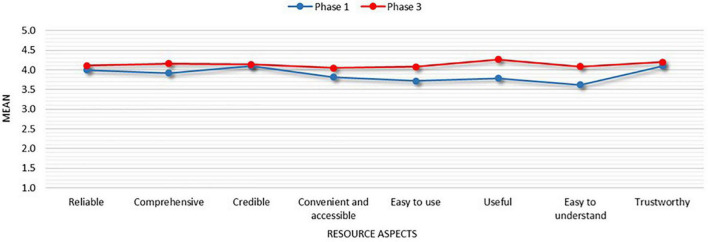
Mean values of key aspects of academic integrity resources before (phase 1) and after (phase 3) implementation of induction tasks.

As described in the Section “Materials and methods,” for comparative purposes, students participating in phases 1 and 3 were asked to rate the same eight aspects of academic integrity resources (see Section “Research design”) on a Likert scale from 1 (completely disagree) to 5 (completely agree). Such aspects and their ratings before (phase 1) and after (phase 3) the design of the suite of new academic integrity resources are summarised in Table 2 and Figure 3. Because of the time of data collection, participants in phases 1 and 3 were from two different cohorts of students, i.e., different academic years. This is because postgraduate programmes in the UK only last for 1 year. Nevertheless, a steady improvement in all aspects has been observed from the January 2020 (phase 1) to November 2020 questionnaire (phase 3), demonstrating an overall positive reception of the new academic integrity material developed in phase 2.

**TABLE 2:** Mean values of the eight aspects of academic integrity resources before (phase 1) and after (phase 3) implementation of the new academic integrity module.

Aspects of academic integrity resources	Phase 1 (*N* = 39)		Phase 3 (*N* = 130)		Comparison			
	M	SD*	M	SD*	M difference	*p*-value	SE*	t statistic
Reliable	4.00	1.08	4.11	1.28	0.11	0.627	0.226	0.487
Comprehensive	3.92	0.87	4.16	1.28	0.24	0.275	0.219	1.096
Credible	4.10	1.07	4.14	1.30	0.04	0.861	0.228	0.175
Convenient and accessible	3.82	1.12	4.05	1.29	0.23	0.316	0.229	1.005
Easy to use	3.72	0.92	4.08	1.20	0.36	0.086	0.209	1.726
Useful	**3.79**	**1.06**	**4.27**	**1.22**	**0.48**	**0.028**	**0.216**	**2.210**
Easy to understand	**3.62**	**0.96**	**4.09**	**1.24**	**0.47**	**0.031**	**0.216**	**2.168**
Trustworthy	4.10	1.21	4.20	1.30	0.10	0.669	0.234	0.428

In bold are the statistically significant differences. *M, mean; SD, standard deviation; SE, standard error.

All eight aspects scored higher ratings in phase 3. Independent sample *t*-tests with Bonferroni correction performed on the eight aspects showed a statistically significant improvement of the perceived usefulness of the resources (from *M* = 3.79 in phase 1, to *M* = 4.27 in phase 3, *p* = 0.028; *t* = 2.210) and their understandability (from *M* = 3.62 in phase 1 to *M* = 4.09 in phase 3, *p* = 0.031; *t* = 2.168). Aspects of trustworthiness and credibility increased from a *M* = 4.10 to *M* = 4.20 for trustworthiness and *M* = 4.10 to *M* = 4.14 for credibility in phase 1 and phase 3 respectively. Although their increases in phase 3 were not statistically significant, they had been the two highest ranked aspects in phase 1, so the margin for improvement was relatively smaller.

The exploration of changes in perceived usefulness and understandability of the resources from phase 1 to phase 3 in terms of proportion of students rating them 4 (agree) and 5 (completely agree), also showed striking results; in phase 3, in fact, 17.1% more students agreed that the new resources were useful, and 28.5% more students agreed that they were easy to understand.

The vast majority of students (85%) also found the new module adequate in supporting their learning; the remaining 12% of students answered “maybe” and a very small percentage of students (3%) considered the module inadequate. It is notable that this feedback was received from postgraduate students who attend 1-year programmes, and therefore do not have access to previous years’ resources or modules. Furthermore, the study was conducted prior to students’ first assessed submissions, hence, it was not possible to gauge their actual understanding of academic integrity principles.

In the phase 3 questionnaire, students were asked to rate their confidence regarding academic integrity concepts prior to and following the use of the new resources on a Likert scale from 1 (very worried) to 5 (very confident). Results show that only 26% of respondents were confident (rated 4 and 5) before using resources. However, this percentage increased to 60.6% after use. Nevertheless, despite the considerable increase in positive perceptions recorded in phase 3, around 28.3% of respondents still believed resources have the potential to be improved further.

**FIGURE 4:**
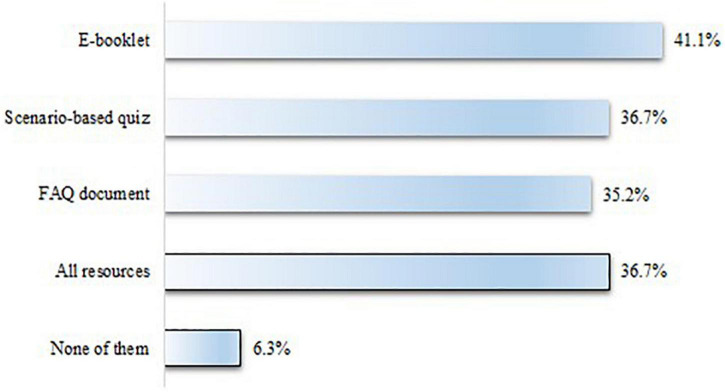
New academic integrity resources as ranked by perceived usefulness (according to student feedback).

In the phase 3 questionnaire, students were asked to indicate which of the new academic integrity resources they found most useful; they could select more than one option when answering this question (Figure 4).

All resources were well-received; however, the e-booklet scored particularly highly (41.1%), perhaps due to the attractiveness of its innovative format, one not normally used to deliver academic integrity content; this was followed by the scenario-based 20-question quiz (36.7%) and the FAQ document (35.2%). A small percentage of students (6.3%) reported none of the resources as useful. However, this could be due to the poor familiarity, at such an early stage of their studies, with the platform (Blackboard) in which they are embedded. Live online sessions were not included in the list, as the questionnaire was distributed before the sessions took place. Nevertheless, their usefulness was demonstrated by the consistently high number of students (about half of the full cohort in each one) attending them.

### Analysis of open-text data from phase 3 questionnaire

Open-text questions were included in the phase 3 questionnaire, allowing students to elaborate on their view of the new resources and provide suggestions for future improvement. Qualitative data suggests that students highly valued the resources provided to them, which helped them develop an enhanced understanding of academic integrity concepts and varied types of breaches in academic integrity:

“I didn’t expect to get such a detailed (and not boring) introduction on this subject.” (Participant 28, Switzerland, Male)

“I have attended lectures of [anonymised], their experience with students who used unfair means (knowingly or unknowingly) helped me a lot. Before that, I know just the theory behind unfair means but after examples, I realised that I do not know much about unfair means in practice.” (Participant 114, India, Male)

### Structured and accessible academic integrity materials

Two main themes were identified in qualitative data, corresponding to the Academic Integrity Model (Bretag et al., 2014): aspects of access and support of academic integrity resources. Students reported that having all resources in one virtual space was very helpful. For example, having a single online module covering academic integrity throughout their study allowed them to learn about these concepts at the beginning of their studies, as well as throughout the academic year and at critical times, such as before coursework submission periods:

“Everything I need to establish what falls or doesn’t under Academic Integrity can be found in the folders; which has been helpful.” (Participant 31, Netherlands, Female)

“I appreciated the introductory material, and know where to go for more information/guidance when I need it.” (Participant 37, UK, Male)

However, students also expressed a preference towards a structure of the resources that avoids information repetition or cognitive overload. Most suggestions for improvement related to making resources organised in a clear fashion on Blackboard. Many students who had not used Blackboard prior to joining the university found it challenging to navigate and struggled to follow material, particularly at the beginning of the academic year. Student recommendations included walkthrough videos, explaining the overall structure of resources, brief videos with clearly labelled topics, and a navigation side panel in the online space:

“The Blackboard software can be overwhelming as a new user. Clearer signposting to resources in general may improve access to learning materials.” (Participant 8, UK, Male)

“They are fine, though I think it needs to be streamlined a bit. There is a lot of content, and some of it is a bit repetitive. There should just be an explanatory video and then the quiz.” (Participant 23, UK, Male)

“I guess creating the whole information in form of some movie, while sticking only to the topic, would be more beneficial. And keeping the length of the videos small or making different videos for each topic would make it easy to search in hour of need. E.g., a student might suddenly get confused about something and search for an answer, but for that he’ll have to go through the whole video.” (Participant 29, India, Female)

These recommendations will be taken into consideration in future modifications of the resources.

### Engaging contents and mixed resources formats

When considering the format of academic integrity resources and support, students welcomed the variety in means of delivery (videos, e-booklets, gamified quizzes with cartoons, FAQ, and online drop-ins) finding them particularly engaging. Students made very positive comments regarding the online quizzes, which contain cartoon scenarios stories based on real cases. According to students, real examples support comprehension of academic integrity concepts better, connecting them with practices and the creative use of cartoons enhanced student engagement:

“Gave overview of what unfair means (academic integrity) are the quiz to test our understanding–made it more fun, and meant it wasn’t just documents that we had to read.” (Participant 27, UK, Female)

“I particularly liked the quiz as it made me reread things in depth to get the right answer and it was real life examples rather than just pages of theory.” (Participant 44, UK, Female)

“I liked the idea of presenting doodles in each question. I never felt bored because of them and I think I can use this idea of creating doodles.” (Participant 25, India, Male)

Physical copies and printable versions were also frequently mentioned by students in comments. Although students preferred the mixed format provided by online resources, they suggested that physical copies, or downloadable materials, should be made available for students as an additional option:

“Email some of the documentation out because for the first couple of weeks I was still trying to get the hang of Blackboard and its multiple folders/files.” (Participant 31, Netherlands, Female)

“The booklet of plagiarism is only available online, it’s better if we can download it.” (Participant 28, China, Female)

This goal could be easily achieved by sending regular email reminders to students with links to online resources and PDF attachments.

## Discussion

This manuscript adopted a pedagogical approach to promoting academic integrity to students in higher education (Walker and White, 2014; Richards et al., 2016). The study aimed to showcase the design process and evaluate the effectiveness of a newly created academic integrity module in relation to enhancing student understanding of academic integrity concepts, policies, and practices. Tailored to students’ needs (Macfarlane et al., 2014), a sequential three-phase study was undertaken, collecting initial student feedback (phase 1), implementing an online academic integrity module (phase 2), and conducting a post-launch evaluation survey (phase 3). The project showed strong potential in supporting the development of students’ academic integrity.

Consistent with existing literature, this research found that, compared with home students, international students are less likely to be informed about academic integrity policies before their arrival in academic departments (Bista, 2011; Fatemi and Saito, 2020). However, both home and international students participating in the study expressed a desire for academic departments to improve academic integrity resources, rather than using punishment and threats to prevent breaches. The results of this study provide supporting evidence for the arguments of previous studies, which argue that an educative approach to academic integrity is more effective than punitive measures (Miller et al., 2011; Palmer et al., 2019; Sun and Hu, 2020).

This study also highlights the importance of an early educative intervention to support the academic integrity of students, particularly with programmes with diverse student cohorts. The findings of this research revealed that a main barrier to student understanding of academic integrity is definition-driven terminologies that lack concrete examples. This finding is consistent with the literature, suggesting that universities should avoid complex language related to academic integrity (Bretag et al., 2019) and go beyond providing statements and definitions of academic misconduct (Risquez et al., 2013).

Another barrier reported by participants is a lack of creative resources and an absence of supportive tone from academic departments; this discourages student engagement and understanding of academic integrity; this broadly corresponds to findings of Bertram Gallant (2008), who stressed the importance of creating a supportive learning-oriented environment which fosters development in students’ academic integrity.

According to the findings of this research, an online academic integrity module has proven to be an effective intervention strategy for increasing student awareness and understanding of academic integrity related concepts, procedures, and policies. This is consistent with the literature suggesting that academic integrity education programmes can positively influence student attitudes and reduce breaches of academic integrity (Greer et al., 2012; Obeid and Hill, 2017; Levine and Pazdernik, 2018; Sefcik et al., 2019). As previously reported, there are relatively few empirical studies directly addressing academic integrity through both the design and evaluation of intervention strategies. This manuscript fills this important gap in the literature, showing both the design and evaluation of the online module, such as how the design of the module was informed by student feedback and how the module promoted academic integrity among students (Stoesz and Yudintseva, 2018; Perkins et al., 2020). Student participants in the research reported a strong preference towards academic integrity resources and support that are structured and easy to access at different stages of their study. Comparative data analysis revealed the online academic integrity module significantly enhanced various aspects of academic integrity resources, particularly aspects of usefulness and comprehension of student perspectives. Results also revealed a remarkable increase in students’ confidence regarding their knowledge of concepts of academic integrity. This evidences the importance of establishing early intervention, and a continuously accessible online course that promotes academic integrity in higher education.

Findings also highlight the importance of the use of a variety of media when delivering academic integrity resources to enhance student engagement and understanding. Students showed a preference towards online academic integrity booklets with examples of detailed analysis of good and poor practice; this allowed them to learn about different types of breaches. This concords with the findings of Boehm et al. (2009), who showed clear examples of what constitutes unethical academic behaviour are able to help prevent academic integrity breaches. Furthermore, students appreciated cartooned scenarios, entailing storytelling, and praised the entertaining and relatable nature of these materials. Results here reflect those of Macfarlane et al. (2014), who highlighted the need to use engaging techniques, such as storytelling and narration, to teach academic integrity related topics.

### Limitations and future directions

The results of this study have important implications for higher education with respect to the design of academic integrity resources to support students’ academic transition. As participants to this research were postgraduate students with essay-based assignments of social science subjects, generalisability of the results is limited. A second limitation regards the possibility of response bias in research and the associated implications. Participating students knew that the authors sought their feedback to evaluate a newly developed module, and they may have wished to please them and give them what they thought they expected (i.e., positive perceptions of the new materials). The authors attempted to minimise this issue by asking a research assistant to moderate the focus group. However, it would have been impossible to minimise this possibility. A further limitation regards the small sample size of the phase 1 study, which may also contribute to reducing the generalisability of the results. Future research may seek to focus on different subject disciplines, and compare results with the current study. Additionally, further studies may also explore the impact of the new module on students’ long-term engagement with academic integrity practices.

## Data availability statement

The original contributions presented in this study are included in the article/Supplementary material, further inquiries can be directed to the corresponding author.

## Ethics statement

The project has received ethics approval (ID: 032172) from The University of Sheffield Ethics Committee. An information sheet was provided to participants prior to data collection. Inform consent was collected prior to the survey and interviews.

## Author contributions

Both authors listed have made a substantial, direct, and intellectual contribution to the work, and approved it for publication.
